# Synergistic antibiofilm efficacy of a gallotannin 1,2,6-tri-O-galloyl-β-D-glucopyranose from *Terminalia chebula* fruit in combination with gentamicin and trimethoprim against multidrug resistant uropathogenic *Escherichia coli* biofilms

**DOI:** 10.1371/journal.pone.0178712

**Published:** 2017-05-31

**Authors:** Anwesa Bag, Rabi Ranjan Chattopadhyay

**Affiliations:** Agricultural and Ecological Research Unit, Indian Statistical Institute, Kolkata, India; Laurentian, CANADA

## Abstract

In recent years the emergence of multiple drug resistance microbes has become a global public health problem. The aim of the present investigation was to evaluate possible antibiofilm efficacy of a gallotannin 1,2,6-tri-O-galloyl-β-D-glucopyranose from *Terminalia chebula* fruits alone and in combination with gentamicin and trimethoprim against preformed biofilms of multidrug-resistant (MDR) uropathogenic *E*. *coli* isolates using microbroth dilution, checkerboard titration and kill kinetics methods. Test gallotannin showed > 50% antibiofilm efficacy after 24 h when administered alone whereas gentamicin and trimethoprim failed to do so. But in combination, test gallotannin/gentamicin and test gallotannin/trimethoprim showed 71.24±6.75% and 93.4±8.46% antibiofilm activity respectively. On the basis of FICI values, test gallotannin/gentamicin showed synergistic interactions against 71.42% and test gallotannin/trimethoprim against 85.71% biofilm forming test bacterial isolates. Kill-kinetics study confirmed their synergistic interactions. Thus, gentamicin and trimethoprim in combination with test gallotannin may have potential for treatment of urinary tract infections caused by biofilm forming MDR uropathogenic *E*. *coli*.

## Introduction

In recent years the emergence of multidrug resistance microbes has become a global public health problem. Despite the recent introduction of highly potent newer antibiotics such as the aminoglycosides, fluoroquinolones and the third generation of cephalosporins into clinical practice, multidrug-resistant pathogens pose a major therapeutic problem for clinicians worldwide [1]. This drug resistance problem is considered to be associated mostly by their biofilm forming ability [2].

Bacterial biofilms are surface associated communities of cells embedded in a self-produced matrix. This matrix consists of extracellular polymeric substances (EPS) and it is one of the most important factors contributing to increase tolerance to antibiotics associated with bacterial biofilms [3]. Bacteria embedded in biofilms are less susceptible to host defenses and more resistant (10–1000 times) to antimicrobial products than their planktonic counterparts [4]. According to the National Institutes of Health (NIH), biofilm forming bacteria involved up to 80% of all infections [5]. In drug resistant bacteria, the antibiotic concentration necessary to eliminate biofilms often exceeds the highest deliverable concentration of antibiotics compared to planktonic bacteria which in subsequent rendered the conventional antibiotics ineffective against the biofilm-forming drug-resistant microbes [6].

Among the biofilm forming bacterial pathogens, *Escherichia coli* is the most common biofilm-forming bacterial species in urinary tract infections. It is also frequently responsible for other types of infections in our body [7]. In recent years, the emergence of multidrug resistant (MDR) uropathogenic *E*. *coli* has become a global public health problem. Most of the conventional antibiotics rendered ineffective against this bacterial pathogen. This problem demands that an alternative approach should be made to make the conventional antibiotics effective against the MDR *E*. *coli* by augmenting their antimicrobial efficacy.

Our previous study on combination effects of test gallotannin 1,2,6-tri-O-galloyl-β-D-glucopyranose with selected antibiotics (amoxicillin, ceftazidime, ciprofloxacin, gentamicin and trimethoprim) against planktonic cells of MDR uropathogenic *E*. *coli* isolates revealed that test gallotannin exhibited synergistic antibacterial activity against the studied bacteria in combination with gentamicin and trimethoprim and additive effects with amoxicillin, ciprofloxacin and ceftazidime [8]. In the present investigation, an attempt has therefore been made to evaluate whether this synergistic antibacterial activity of test gallotannin in combination with antibiotics gentamicin and trimethoprim against MDR uropathogenic *E*. *coli* is associated with their antibiofilm activity or not.

## Materials and methods

### Test compound

**Fig 1:**
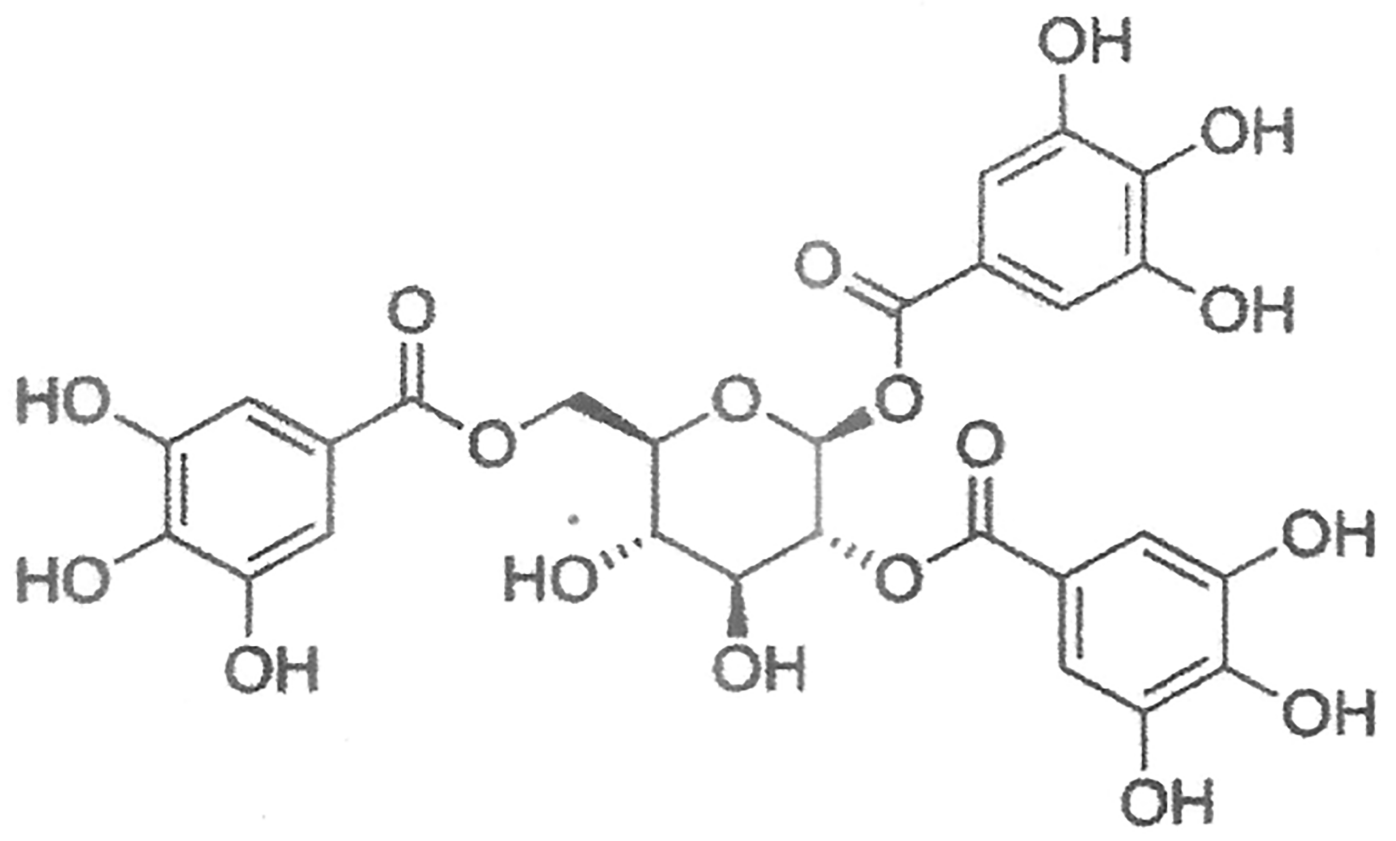
Chemical structure of test gallotannin (1,2,6-tri-O-galloyl-β-D-glucopyranose).

A gallotannin, 1,2,6–tri-O-galloyl-β-D-glucopyranose (Fig 1) was isolated from hydro ethanol extract of *T*. *chebula* fruits by bioassay-guided fractionation and characterized by extensive spectroscopic analyses (FT-IR, ^1^H-NMR, ^13^C-NMR and ESI-MS) [8]. This isolated gallotannin (Fig 1) was used in the present investigation.

### Microorganisms

Seven multidrug-resistant clinical isolates of *Escherichia coli* from urine samples collected from patients having indwelling catheter for more than two weeks, and urine microscopy shows pyuria were used in the present investigation. Isolates were kindly provided by the Department of Microbiology, NRS Medical College and Hospital, Kolkata, India. Their antibiotics resistance profiles are summarized in Table 1. Internal quality assurance was ensured using reference *E*. *coli* (ATCC 8739) strain procured from National Chemical Laboratory, Pune, India. The isolates were maintained on nutrient agar slants at 4°C.

**Table 1:** Bacterial strains used and their resistance profile.

Organism	Type	Resistance profile
***E*. *coli* (EC1601)**	**MDR**	**GM**^**R**^, **CIP**^**R**^, **TMP**^**R**^
***E*. *coli* (EC1602)**	**MDR**	**CEF**^**R**^, **GM**^**R**^, **TMP**^**R**^, **NOR**^**R**^
***E*. *coli* (EC1603)**	**MDR**	**IMP**^**R**^, **GM**^**R**^, **TMP**^**R**^, **CIP**^**R**^
***E*. *coli* (EC1604)**	**MDR**	**CEF**^**R**^, **NOR**^**R**^, **IMP**^**R**^, **GM**^**R**^
***E*. *coli* (EC1605)**	**MDR**	**GM**^**R**^, **CEF**^**R**^, **TMP**^**R**^
***E*. *coli* (EC1606)**	**MDR**	**GM**^**R**^, **TMP**^**R**^, **NOR**^**R**^, **IMP**^**R**^
***E*. *coli* (EC1607)**	**MDR**	**CEF**^**R**^, **IMP**^**R**^, **TMP**^**R**^, **GM**^**R**^
***E*. *coli* (ATCC 8739)**	**Type strain**	**----------------------------**

MDR: Multidrug-resistant; CEF: Cefotaxime; CIP: Ciplofloxacin; GM: Gentamicin; IMP: Imipenem; NOR: Norfloxacin; TMP: Trimethoprim; R: Resistant

### Inoculum preparation

The inoculum size of the test bacterial isolates was standardized following the National Committee for Clinical Laboratory Standards guidelines [9]. The bacterial isolates were inoculated in Mueller Hinton Broth (MHB) (Hi-media, Mumbai, India) and incubated at 37°C in a shaker water bath for 3–6 h until the culture attained a turbidity of 0.5 McFarland unit. For experimental purposes, inoculum size of test isolates was adjusted to 5 × 10^5^ CFU/ml.

### Determination of minimum inhibitory concentration (MIC)

Microbroth dilution susceptibility test was performed for minimum inhibitory concentration (MIC) determination in flat-bottom 96-well microtiter plates containing MHB medium (90 μl) in each well. The test compound reconstituted in 0.5% dimethyl sulphoxide (DMSO) was diluted twofold serially with MHB ranging from 2 μg/ml to 512 μg/ml. 100 μl of diluted solution was given in each well containing broth. 10 μl of working inoculum suspension (5 × 10^5^ CFU/ml) was added to the wells. A number of wells were reserved in each plate for control of sterility (no inoculum added), inoculums viability (no sample solution added) and DMSO inhibitory effect. The plates were then incubated for 18 h at 37°C. After incubation, 10 μl of Alamar Blue was added in each well and further incubated for 4 h for a colour change from blue to pink. A blue colour in the well was interpreted as no growth, and a pink indicated growth. MICs were determined as the lowest concentration of the drug that prevented the colour change from blue to pink [10]. Each experiment was repeated thrice.

### Determination of antibiofilm activity of test gallotannin and antibiotics alone and in combination

Biofilm formation was achieved by aliquoting 100 μl of test *E*. *coli* culture (5 × 10^5^ CFU/ml) into wells of 96-well microtiter plate. The plate was incubated for 72 h at 37°C to allow biofilm formation. After incubation, supernatant was removed and each well was washed thoroughly with sterile distilled water to remove free-floating cells; thereafter plates were air-dried for 30 min and the biofilm formed was stained with 100 μl of 0.1% aqueous solution of crystal violet (CV) and kept for 15 min at room temperature. After staining, the excess of stain was removed washing the plate three times with sterile distilled water. Finally, the dye bound to the cells was solubilized by adding 200 μl of 95% ethanol to each well and after 15 min of incubation, absorbance was measured at 595 nm. CV absorbance of all test isolates were found to be ≥1.5 which indicates that the test bacterial isolates have high biofilm forming ability [11]. After biofilm formation, 100 μl of test gallotannin and antibiotics alone and in combination (1:1) at their MIC doses were given in wells of microtiter plate. Well containing equal volume of DMSO (0.5%) in place of test antimicrobials served as control. Plates were further incubated at 37°C for 24 h. Absorbance of the control and treated wells (OD_595nm_) were determined and percent inhibition of biofilm biomass of test gallotannin and antibiotics alone and in combination were calculated using the following formula [12]. Each experiment was repeated thrice.

$$\%\text{ Inhibition of biofilm biomass}=\;\frac{{\text{OD}}_{\text{Control}}-{\text{ OD}}_{\text{Treatment}}}{{\text{OD}}_{\text{Control}}}\;\times 100$$

### Determination of fractional inhibitory concentration index (FICI)

For determining FICI values, effects of test gallotannin and antibiotics alone and in combination on MDR uropathogenic *E*. *coli* biofilms was evaluated using the method of Ramage et al. (2001) [13]. Biofilm formation of test bacterial strains was achieved following the same protocol as described above. After biofilm formation, the medium was aspirated gently and non-adherent cells were removed by washing the biofilms three times with sterile PBS. Then 100 μl of 2-fold serial dilutions (1/32 × MIC to 4 × MIC) of test gallotannin and antibiotics were added to each biofilm containing wells and microtiter plates were incubated for 24 h at 37°C. Minimum biofilm eradication concentrations (MBECs) were determined by the XTT reduction assay following the method of Ramage et al. (2001) [13]. FIC_indices_ were calculated using the formula: FIC_index_ = (MBEC of antibiotic in presence of test gallotannin/MBEC of antibiotic alone) + (MBEC of test gallotannin in presence of antibiotic/MBEC of test gallotannin alone). The results were interpreted according to FIC_indices_ as follows: ‘synergy’ (FICI ≤ 0.5), ‘additive’ (FICI > 0.5–4) and ‘antagonism’ (FICI > 4) [14]. All the experiments were repeated thrice.

### Kill kinetics study

To evaluate the type of antibiofilm interactions (synergistic, additive or antagonistic) of test gallotannin in combination with antibiotics, time-kill assay was performed. Here, the combination of ¼ × MBEC was applied [14, 15]. For this, biofilms of most susceptible test isolate [*E*. *coli* (EC1606)] were allowed to form in 96-well microtiter plates following the same procedure as described above and challenged with test gallotannin and antibiotics alone and in combination (1:1) at their ¼ MBEC doses. Microtiter plates were incubated at 37°C for 24 h. Viable cells were measured over a series of time intervals (0, 2, 4, 6, 8 and 24 h) and plotted. Each experiment was repeated thrice. Synergy was defined as > 100-fold or >2log_10_ decrease in colony count at 24 h by the combination when compared with their single agent [14].

The types of interactions were considered as follows.

‘synergistic’: > 2 log_10_ CFU/ml decrease; ‘additive’: < 2 log_10_ CFU/ml decrease; and ‘antagonistic’: > 2 log_10_ CFU/ml increase in bacterial colony count at 24 h by combination treatment in comparison with the killing by the most active single agent [14, 15].

### MTT assay using human normal colon cell line

#### Cell culture

Human normal colon cell line was obtained from American Type Culture Collection (ATCC, USA) and maintained in EMEM medium which was supplemented with 10% fetal bovine serum (FBS), penicillin (100 units/ml), streptomycin (100 μg/ml) and 1% sodium pyruvate. The cells were incubated at 37°C in a humidified 5% CO_2_ incubator.

#### MTT assay

Cytotoxic potential of test galotannin that showed synergistic interactions was tested in triplicate by MTT [3-(4,3-dimethylthiazole-2-yl)-2,5-diphenyltetrazolium bromide] assay [16] using human normal colon cell line (CCD-18Co) with slight modification. Briefly, after being harvested from culture flasks the cells (100μl) were seeded at a density of 1 × 10^5^ cells/ml in each well of 96 well plate containing 100 μl of fresh growth medium per well and cells were permitted to adhere for 24h at 37°C. The medium was removed after 24 h of incubation and 100 μl of fresh medium containing different concentrations (2 μg/ml—512 μg/ml) of test gallotannin were added. To control wells only culture medium (100μl) was used. Following 72 h of incubation, 20 μl of MTT (5 mg/ml) was added into each well and further incubated for another 4h. The formation of insoluble purple formazan from yellowish MTT by enzymatic reduction was dissolved in DMSO (100 μl) after removal of medium. The plates were shaken for 5 min and the absorbance was measured in a microplate reader at 570 nm with 630 nm as reference wavelength. The percent cell inhibition was determined using the following formula:

$$\%\;\text{Cell inhibition}=100-\;\frac{\text{Absorbance of treated cells}}{\text{Absorbance of control cells}}\times 100$$

A dose-response curve was plotted from which IC_50_ was calculated.

### Statistical analysis

Data were statistically analysed using SPSS software: Version 18.0. Two-way analysis of variance (ANOVA) and Student’s t-test were applied for analysis of data with a level of significance set at p < 0.05.

## Results

The bacterial strains used in the study and their resistance profile are shown in Table 1.

MIC values of test gallotannin and antibiotics gentamicin and trimethoprim against the studied bacteria are cited in Table 2. The MIC values of test gallotannin, gentamicin and trimethoprim against the test clinical isolates were found to be 14.09 ± 3.49 μg/ml, 60.95 ± 9.62 μg/ml and 30.47 ± 4.81 μg/ml respectively whereas these values against type strains were 10.66 ± 4.61 μg/ml, 26.66 ± 9.23 μg/ml and 13.33 ± 4.61 μg/ml respectively (Table 2).

**Table 2:** Minimum inhibitory concentration (MIC) values of test gallotannin and antibiotics gentamicin and trimethoprim against planktonic cells of multidrug- resistant uropathogenic *E*. *coli*.

Microorganisms	TG	GM	TMP
*E*. *coli* (ATCC 8739)	10.66 ± 4.61	26.66 ± 9.23	13.33 ± 4.61
*E*. *coli* (EC1601)	13.33 ± 4.61	53.33 ± 18.47	26.66 ± 9.23
*E*. *coli* (EC1602)	16.00 ± 0.00	64.00 ± 0.00*	26.66 ± 9.23
*E*. *coli* (EC1603)	13.33 ± 4.61	53.33 ± 18.47	32.00 ± 0.00*
*E*. *coli* (EC1604)	13.33 ± 4.61	64.00 ± 0.00*	32.00 ± 0.00*
*E*. *coli* (EC1605)	13.33 ± 4.61	64.00 ± 0.00*	32.00 ± 0.00*
*E*. *coli* (EC1606)	16.00 ± 0.00	64.00 ± 0.00*	32.00 ± 0.00*
*E*. *coli* (EC1607)	13.33 ± 4.61	64.00 ± 0.00*	32.00 ± 0.00*

Values are mean ± SD of triplicate experiment

*p < 0.05 in comparison with type strain

**Fig 2:**
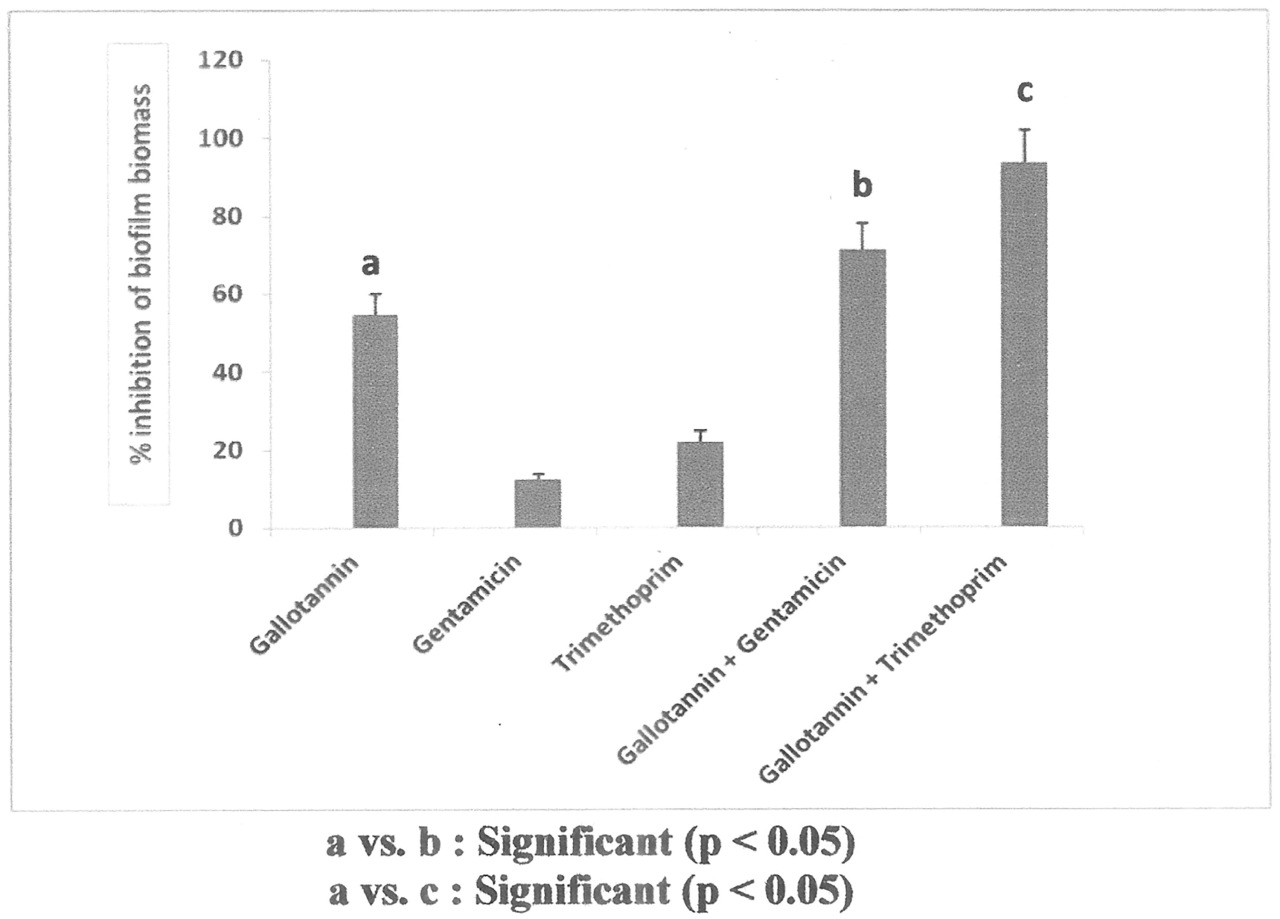
Antibiofilm efficacy of test gallotannin and antibiotics gentamicin and trimethoprim alone and in combination against MDR uropathogenic *E*. *coli* biofilms.

TG: Test gallotannin; GM: Gentamicin; TMP: Trimethoprim;

After determining the MIC values of test samples against the studied bacteria, their alone as well as combination effects on biofilms produced by the test bacterial isolates were observed. The results of antibiofilm efficacy of test samples alone and in combination are shown in Fig 2. From Fig 2, it was observed that the test gallotannin showed 54.60 ± 5.27% inhibition on biofilm biomass of test bacterial isolates whereas these values for gentamicin and trimethoprim were 12.50 ± 1.21% and 21.87 ± 2.84% respectively after 24 h of administration. But test gallotannin/ gentamicin and test gallotannin/ trimethoprim combinations showed 71.24 ± 6.75% and 93.40 ± 8.46% inhibition on biofilm biomass respectively.

Table 3 shows the FICI values of test gallotannin/ gentamicin and test gallotannin/ trimethoprim combinations on biofilms formed by the studied bacteria. It was observed that both test gallotannin/ gentamicin (FICI: 0.156–0.375) and test gallotannin/ trimethoprim (FICI: 0.093–0.50) combinations exhibited synergistic antibiofilm activity against most of the studied bacteria.

**Table 3:** Fractional inhibitory concentration values (FICI) of test gallotannin in combination with antibiotics gentamicin and trimethoprim against MDR uropathogenic *E*. *coli* biofilms.

Microorganism	Combinations					
	TG + GM			TG + TMP		
	FIC	FICI	Remarks	FIC	FICI	Remarks
***E*. *coli (EC1601)***	0.250 (TG)	0.375	S	0.031 (TG)	0.093	S
	0.125 (GM)			0.062 (TMP)		
***E*. *coli (EC1602)***	0.125 (TG)	0.250	S	0.062 (TG)	0.312	S
	0.125 (GM)			0.250 (TMP)		
***E*. *coli (EC1603)***	0.250 (TG)	1.25	ADD	0.062 (TG)	0.124	S
	1.00 (GM)			0.062 (TMP)		
***E*. *coli (EC1604)***	0.031 (TG)	0.156	S	0.500 (TG)	1.00	ADD
	0.125 (GM)			0.500 (TMP)		
***E*. *coli (EC1605)***	0.125 (TG)	0.625	ADD	0.25 (TG)	0.50	S
	0.500 (GM)			0.25 (TMP)		
***E*. *coli (EC1606)***	0.031 (TG)	0.281	S	0.062 (TG)	0.187	S
	0.250 (GM)			0.125 (TMP)		
***E*. *coli (EC1607)***	0.250 (TG)	0.375	S	0.125 (TG)	0.250	S
	0.125 (GM)			0.125 (TMP)		

TG: Test gallotannin; GM: Gentamicin; TMP: Trimethoprim

S: Synergistic; ADD: Additive

**Fig 3:**
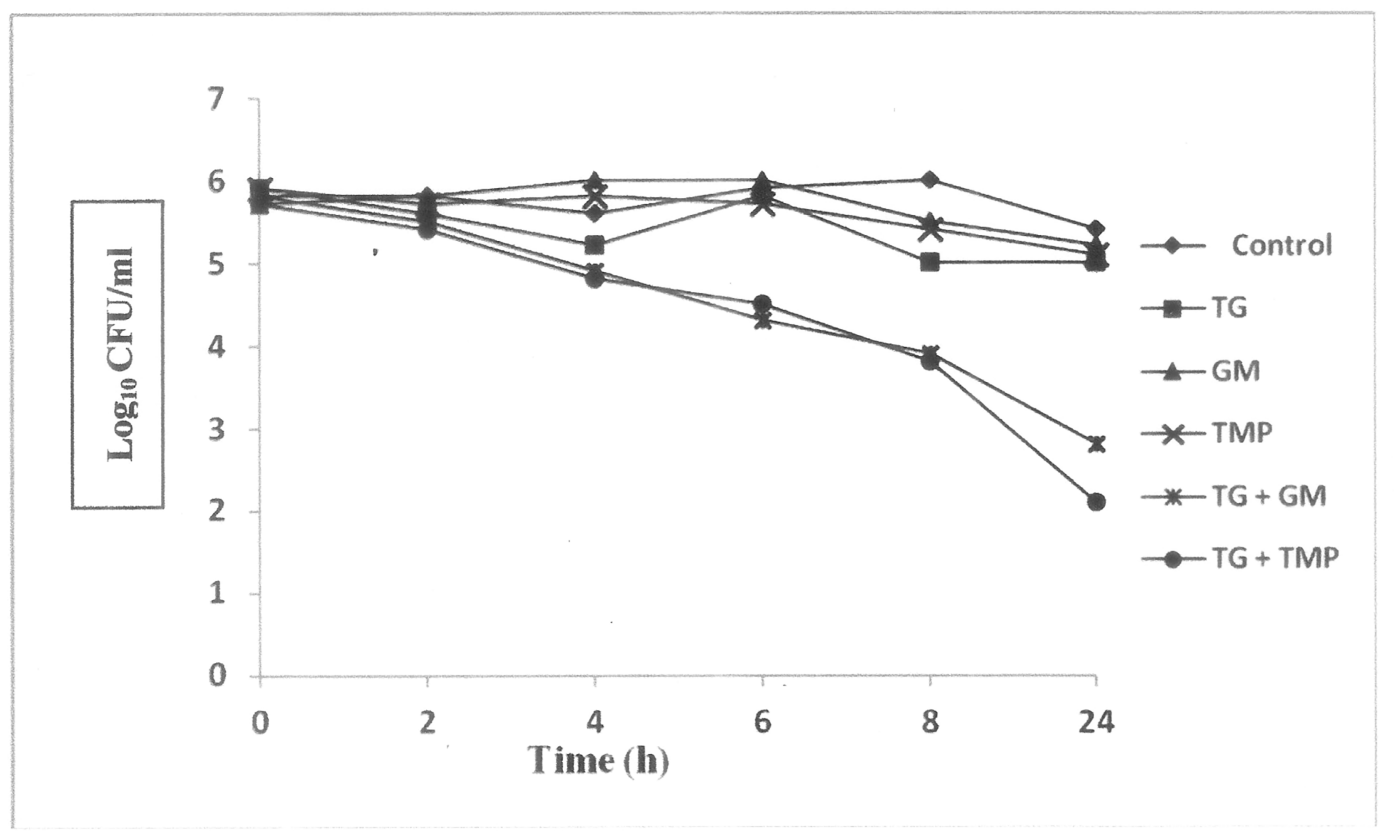
Kill kinetics curves on antibiofilm efficacy of test gallotannin and antibiotics gentamicin and trimethoprim alone and in combination against uropathogenic MDR *E*. *coli* biofilms. [TG: Test gallotannin; GM: Gentamicin; TMP: Trimethoprim].

Fig 3 shows the results of kill-kinetics study of test gallotannin and antibiotics alone and in combination against the studied bacterial biofilms. It was observed that a reduction > 2 Log_10_ CFU/ml decrease in colony count was observed in both test gallotannin/ gentamicin and test gallotannin/ trimethoprim combinations in comparison with the killing by the most active single agent.

The results of MTT assay revealed that test gallotannin failed to show any cytotoxic effect on human normal colon cell line at recommended dosage level and the IC_50_ value was found to be > 512 μg/ml (Table 4).

**Table 4:** Cytotoxic potential of test galloatannin 1,2,6–tri-O-galloyl-β-D-glucopyranose using MTT assay.

Concentration (μg/ml)	% cell death (72 h)	IC_50_ (μg/ml)
0	0	> 512
2	0	
4	0	
8	0	
16	0	
32	0	
64	7.86 ± 0.85	
128	11.70 ± 1.05	
256	17.4 ± 1.21	
512	21.7 ± 1.16	

Values are mean ± SD of triplicate experiments

## Discussion

Synergistic combinations of antimicrobials have been introduced in recent years as more successful strategies to combat infections involving MDR bacterial pathogens. Generally, synergistic combinations have proven to be an essential feature of antimicrobial treatment due to a number of important considerations viz. (i) they increase activity through the use of compounds with synergistic activity; (ii) they thwart drug resistance; (iii) they decrease required doses, reducing both cost and adverse/toxic side effects and (iv) they increase the spectrum of antimicrobial activity. Several studies recommend synergistic combination therapy as the treatment of choice in biofilm-associated infections [17, 18].

In the present investigation, to evaluate possible synergistic antibiofilm efficacy of test gallotannin against MDR uropathogenic *E*. *coli* biofilms with conventional antibiotics gentamicin and trimethoprim that were rendered ineffective against these studied clinical bacterial isolates, at first MIC values of these test antimicrobials against planktonic cells of MDR uropathogenic *E*. *coli* isolates were determined. It was observed that at their alone effects the MIC values of test gallotannin against studied clinical bacterial isolates were much lower than the MIC values of gentamicin and trimethoprim. Besides, there was no significant difference in MIC values of test gallotannin against test clinical isolates compared to type strains. But the MIC values of both gentamicin and trimethoprim were significantly (p < 0.05) different between clinical isolates and type strains (Table 2).

Using the MIC values of test antimicrobials, their antibiofilm efficacy alone and in combination against the studied bacterial biofilms were evaluated. A reduction in > 50% in biofilm biomass by test antimicrobials was considered as active. When administered individually, the test gallotannin showed > 50% inhibition in biofilm biomass whereas gentamicin and trimethoprim failed to show > 50% antibiofilm efficacy after 24 h of administration. But in combination with test gallotannin both gentamicin and trimethoprim showed synergistic antibiofilm efficacy. These findings suggest that gallotannin has the ability to inhibit biofilm biomass formed by studied bacteria and also to augment the antibiofilm efficacy of conventional antibiotics gentamicin and trimethoprim (Fig 2).

To evaluate the type of antibiofilm interactions (synergistic, additive or antagonistic), FICI values of test antimicrobials using checkerboard titration method were determined. From FICI values (Table 3), it was observed that test gallotannin/ gentamicin combination showed synergistic antibiofilm efficacy against 71.42% test bacterial isolates whereas this value for test gallotannin/ trimethoprim combination was 85.71%.

In order to confirm the synergistic antibiofilm efficacy of test gallotannin in combination with gentamicin and trimethoprim, kill-kinetics study was performed. A reduction in > 2 Log_10_ CFU/ml decrease in bacterial colony count at 24 h in comparison with the killing by the most active single agent was considered as synergistic [14]. From kill-kinetics curve, it was observed that test gallotannin/ gentamicin and test gallotannin/ trimethoprim combinations decreased the bacterial colony count by 2.2 Log_10_ CFU/ml and 2.9 Log_10_ CFU/ml respectively at 24 h compared to most active single agent test gallotannin (Fig 3) confirming the synergistic antibiofilm efficacy of test gallotanin with antibiotics gentamicin and trimethoprim against the studied bacterial biofilms. Our results corroborate with the findings of other workers [19, 20] where they found synergistic antibiofilm activity of plant extracts and/ or phytocompounds with conventional antibiotics against disease-producing bacterial and fungal biofilms. Possible mechanisms behind the synergistic interactions of test gallotannin in combination with gentamicin and trimethoprim is not clear right now. Studies have shown that two different anti-biofilm mechanisms are able to modulate biofilm formation: inhibition of bacterial surface attachment and interruption of quorum sensing (QS) [21]. Whether synergistic anti-biofilm activity of test gallotannin in combination with antibiotics gentamicin and trimethoprim is due to inhibition of bacterial surface attachment or interruption of quorum sensing that needs to be investigated.

Furthermore, MTT assay is a rapid and high accuracy colorimetric approach that widely used to determine cell growth and cytotoxicity, particularly in the development of new drug. It measures cell membrane integrity by determining mitochondrial activity through enzymatic reaction on the reduction of yellow tetrazolium MTT to a purple formazan. So the amount of formazan produced reflected the number of metabolically active viable cells [22]. The cytotoxic effect of test gallotannin was investigated *in vitro* on human normal colon cell line using MTT assay and found that the test gallotannin failed to show any toxic potential against human normal colon cell line with IC_50_ > 512 μg/ml which suggests that the test gallotannin can be considered as a safe substance ordinarily.

Thus from the foregoing findings, it was observed that the test gallotannin 1,2,6-tri-O-galloyl-β-D-glucopyranose isolated from *T*. *chebula* fruit has the ability to eradicate biofilms produced by MDR uropathogenic *E*. *coli* alone and in combination with conventional antibiotics gentamicin and trimethoprim *in vitro*. Antibiofilm efficacy of test gentamicin and trimethoprim was also significantly enhanced in presence of test gallotannin. The results provide evidence that the test gallotannin alone and in combination with gentamicin and trimethoprim may serve as a potential source for treatment of biofilm-associated urinary tract infections caused by MDR *E*. *coli*. Further studies in *in vivo* systems are required for their practical applications. This report may serve as a footstep on these important aspects.
